# High-Performance Triboelectric Nanogenerator with Double-Side Patterned Surfaces Prepared by CO_2_ Laser for Human Motion Energy Harvesting

**DOI:** 10.3390/mi15111299

**Published:** 2024-10-25

**Authors:** Dong-Yi Lin, Chen-Kuei Chung

**Affiliations:** Department of Mechanical Engineering, National Cheng Kung University, Tainan 701, Taiwan

**Keywords:** triboelectric nanogenerators, CO_2_ laser, cold imprinting, double-sided morphologies, wearable devices

## Abstract

The triboelectric nanogenerator (TENG) has demonstrated exceptional efficiency in harvesting diverse forms of mechanical energy and converting it into electrical energy. This technology is particularly valuable for powering low-energy electronic devices and self-powered sensors. Most traditional TENGs use single-sided patterned friction pairs, which restrict their effective contact area and overall performance. Here, we propose a novel TENG that incorporates microwave patterned aluminum (MC-Al) foil and microcone structured polydimethylsiloxane (MC-PDMS). This innovative design utilizes two PMMA molds featuring identical micro-hole arrays ablated by a CO_2_ laser, making it both cost-effective and easy to fabricate. A novel room imprinting technique has been employed to create the micromorphology of aluminum (Al) foil using the PMMA mold with shallower micro-hole arrays. Compared to TENGs with flat friction layers and single-side-patterned friction layers, the double-side-patterned MW-MC-TENG demonstrates superior output performance due to increased cone deformation and contact area. The open-circuit voltage of the MW-MC-TENG can reach 141 V, while the short-circuit current can attain 71.5 μA, corresponding to a current density of 2.86 µA/cm^2^. The power density reaches 1.4 mW/cm^2^ when the resistance is 15 MΩ, and it can charge a 0.1 μF capacitor to 2.01 V in 2.28 s. In addition, the MW-MC-TENG can function as an insole device to harvest walking energy, power 11 LED bulbs, monitor step speed, and power a timer device. Therefore, the MW-MC-TENG has significant application potential in micro-wearable devices.

## 1. Introduction

With the increasing demand for healthcare wearable devices and self-powered electronic sensors, eco-friendly energy harvesting technology has become a crucial method for converting ambient energy into electricity using various energy conversion principles, such as piezoelectric [1,2], electromagnetic [3,4], thermoelectric [5,6], pyroelectric [7,8], electrostatic [9,10], triboelectric [11,12,13,14], etc. In particular, Wang’s group proposed triboelectric nanogenerators (TENGs) as a means to convert mechanical energy from the environment into electrical energy by coupling contact electrification and electrostatic induction [15,16,17,18]. To date, TENGs have demonstrated superiority and convenience in the fields of human biomechanical energy collection and motion monitoring owing to their significant advantages of high-power density, low frequency, low cost, and ease of fabrication [19,20,21].

In theory, the output of a TENG depends solely on the actual contact area and surface transfer charge density [22]. Controlling the increase in effective contact area through surface engineering is a widely employed strategy to enhance the electrical performance of TENGs [23,24]. In surface morphology control engineering, various micro- and nano-scaled morphological structures have been employed to enhance the electrical performance of TENGs. These structures include nano-pyramids, lines, cubes [25], nanocolumnars [26], micro balloons [27], and microneedles [28]. However, it is common for only one side of the friction pairs to be patterned, which limits the electrical output performance of the TENG. As a result, to further increase the effective contact area, double-sided patterning surface engineering has been proposed in recent years. For example, microcone–microcone [29], microyarn–microyarn [30], nanogrit–nanogrit [31], micropore–nanoparticles [32], nano roughened structure–nanograting [33], or nanopillar–nanopillar [34], as list in Table 1. Nevertheless, the aforementioned nano/microstructures are primarily fabricated using methods, such as lithography [25], etching [25,29], evaporation [29], plasma [33], electrodeposition [34], and chemical functional groups [35]. These methods are high-cost and time-consuming, which limits the further application and development of TENGs.

In this article, we propose a new double-sided patterning approach to enhance the sustainable mechanical-electrical energy conversion, energy storage, and output performance of a novel TENG consisting of aluminum (Al) with microwave (MW) structure arrays and polydimethylsiloxane (PDMS) with microcone (MC) structure arrays. Moreover, they are fabricated using polymethyl methacrylate (PMMA) molds through a green, low-cost, easy-fabrication CO_2_ laser ablation method [36]. The laser writing technique has proven to be a powerful, precisely programmable process, that has been widely applied in energy storage and wearable electronics [28,37]. Two imprinting methods are employed to pattern the friction layers. The WW-Al is created through room temperature imprinting using a separate mold with a shallow hole array; while the MC-PDMS is produced via a rapid prototyping casting process using a mother mold with a deep hole array. To compare the differences in electrical performance output, four different types of TENGs were invested, as listed in Table 2. The electrical performance of open-circuit voltage (*V_oc_*), short-circuit current (*I_sc_*), transfer charge [38] (*Q_sc_*), and current density (*J_sc_*), of four TENGs, follows: flat-flat-TENG < MW-flat-TENG ≈ flat-MC-TENG < MW-MC-TENG. The *V_oc_*, *I_sc_*, and *J_sc_* of the MW-MC-TENG are 141 V, 71.5 µA, and 2.86 µA/cm^2^, respectively. These values represent increases of 54%, 64%, and 64%, compared to the control flat-flat-TENG attributed to the enhanced effective contact surface area. Additionally, the output power of the MW-MC-TENG can reach 34.7 mW, corresponding to a power density of 1.4 mW/cm^2^, when the external resistance is 15 MΩ. Furthermore, the MW-MC-TENG may charge a 0.1 µF capacitor to 2.01 V in 2.28 s and to a maximum voltage of 2.32 V. The MW-MC-TENG, characterized by its significantly smaller cone height and width achievable under lower laser ablation power, demonstrates superior electrical output performance compared to recently reported double-sided patterned TENG devices, as shown in Table 1. Furthermore, its performance exceeds that of the MN–TEG [28], IWA-SMD-TEG [39], and OL-TENG [40], all of which can be also fabricated using CO_2_ laser technology. In the application test, the MW-MC-TENG was utilized as a wearable self-powered sensor, capable of detecting human walking motion, power 11 LED bulks embedded in a shoe, and power an electronic timer. Overall, the MW-MC-TENG demonstrates a highly sensitive response to motion status under different walking speeds and provides a reliable power supply for low-power electronic devices. This indicates that the proposed MW-MC-TENG has significant potential applications in micro wearable devices.

## 2. Materials and Methods

### 2.1. The Material Selection and Fabrication of Friction Pairs

**Figure 1:**
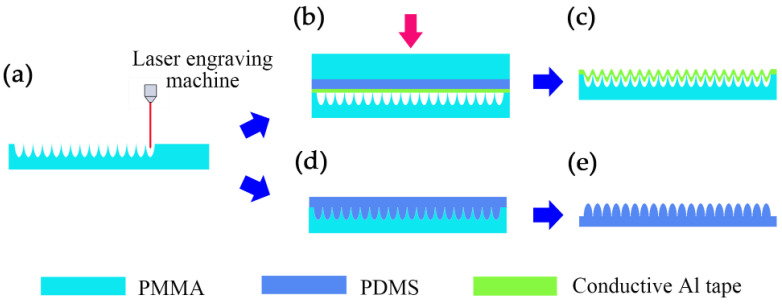
The process of preparing the frictional appearance of MW-MC-TENG. (**a**) Micro-hole arrays were ablated on PMMA molds with a CO_2_ laser machine. (**b**) The Al foil was imprinted into a PMMA mold through a PMMA flat plate and a soft flat PDMS medium under external pressure (the red arrow). (**c**) After removing the PMMA board and PDMS, the positive friction layer adhered to the PMMA mold was formed. (**d**) PDMS was poured into another PMMA mold. (**e**) After baking and demolding, the negative fiction layer was formed.

Preparation of the MW-Al friction and Electrode. The fabrication of the MW-Al electrode is as follows: The PMMA mother mold with micro-pore array was fabricated through CO_2_ laser ablation at a power of 0.06 W, as shown in Figure 1a. CorelDraw software was utilized for the array design, and a CO_2_ laser source (VL-200, General Laser System Inc., Scottsdale, AZ, USA) system was employed to control CO_2_ laser processing parameters. The conductive Al tape with a thickness of 60 µm was affixed to the side of PMMA featuring a microporous array within a 50 mm × 50 mm area. Then, a pre-prepared flat flexible PDMS was sandwiched between a flat PMMA board and the conductive aluminum tape, with pressure applied using a hydraulic press (100 kg/cm^2^) machine, as shown in Figure 1b. The MW-Al tapping on the PMMA played the role of the top tribo-layer and electrode, as shown in Figure 1c.

Preparation of the MC-PDMS film. The PMMA mother mold with a micro-pore array was also fabricated through CO_2_ laser ablation at a laser power of 0.33 W, a scanning speed of 11.4 mm/s, and a PPI of 110 µm, as shown in Figure 1a. Secondly, the PDMS and curing agent were mixed evenly in a 10:1 ratio, and, subsequently, placed into the PMMA mold, as depicted in Figure 1d. The mixture was vacuumed in a vacuum pump kettle for 10 min to ensure that all holes were filled. It was then cured in an oven at 85 °C for 1 h. Finally, the PDMS film was peeled off from the PMMA and cut into sizes of 50 mm × 50 mm, as shown in Figure 1e, to remove irregular burrs. The PDMS films, including flat-PDMS and MC-PDMS, were prepared with identical area (50 mm × 50 mm) and thickness.

### 2.2. The Measurement of MW-MC-TENG

**Figure 2:**
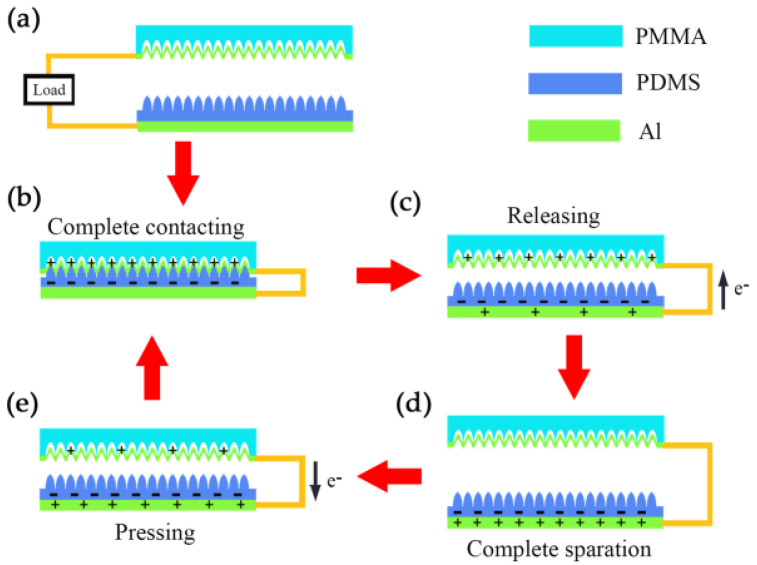
The measurement and working principle of MW-MC-TENG. (**a**) The electrical system of MW-MC-TENG includes the MW-Al friction and conductive layer, the PDMS friction layer, and the Al conductive layer, under the initial state. (**b**) The triboelectric effect with maximum charge transferred, under complete contact. (**c**) The restoring of two contact surfaces induced electronic flow via an external circuit from the bottom electrode to the top electrode when the force is released. (**d**) A new electrical equilibrium at complete separation. (**e**) The electronic flow from the top electrode to the bottom electrode was induced when the pressing again of two contact surfaces.

To test the electrical output performance of the MW-MC-TENG, a vertical pneumatic cylinder (FESTO, D: S-PAZ-DW20-100PPV, Esslingen, Germany) test platform was utilized to facilitate vertical repeated contact-separation of the TENGs. The schematic representation of the electrical system of the MW-MC-TENG is depicted in Figure 2a. The two triboelectric layers of MW-MC-TENG, namely MW-Al and MC-PDMS, were adhered to the drive shaft and the stage, respectively. The two electrodes of MW-MC-TENG consist of flat Al on the stage and MW-Al affixed to the PMMA substrate. The testing platform is driven by air pressure (input air pressure of 2 kg/cm^2^) and operates at a frequency of 3 to 7 Hz, with a force of 30 N, maintaining a distance of 20 mm between the test piece and the MW-Al electrode. The aluminum electrode and the test piece are connected using copper wires to a transient waveform recorder to measure the open-circuit voltage signal during the testing process. When measuring the short-circuit current, it is essential to perform so in parallel with a high-resistance load. Furthermore, to evaluate the various DC performances of MW-MC-TENG, a bridge rectifier was employed to convert the MW-MC-TENG signal, and a capacitor was connected in parallel. Due to its high stability, the platform can maintain stability without degradation during high-cycle impact experiments designed to assess the stability of MW-MC-TENG. The microstructure characterization of MW-MC-TENG was conducted using optical microscopy (OM, Olympus BX 51 M, Tokyo, Japan). The *V_oc_* and *I_sc_* of MW-MC-TENG were measured using an oscilloscope (HIOKI Memory HiCorder MR8870-20, Nagano, Japan).

## 3. Results

### 3.1. The Micromorphology Structure of the MW-Al and MC-PDMS

**Figure 3:**
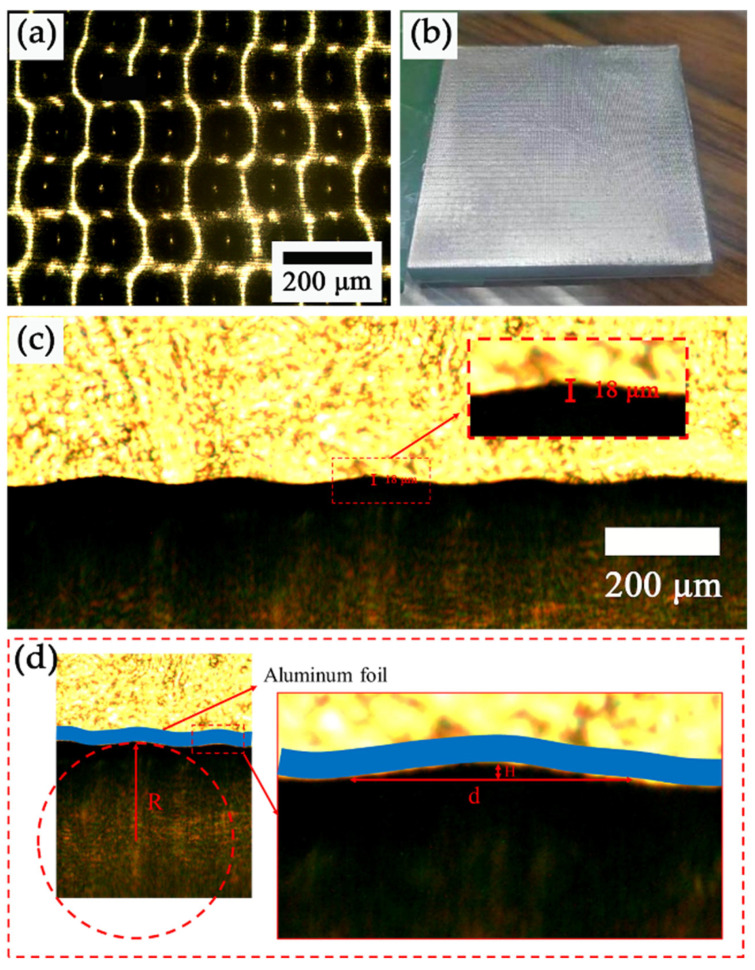
(**a**) Front view OM diagram of the MW-Al foil; (**b**) CCD camera image of MW-Al foil morphology at a distance of 5 cm; (**c**) Side view of the OM diagram of 18 μm microwave structure on Al foil; (**d**) The basic dimension schematics of the MWs.

The micromorphology structure of the MW-Al is illustrated in Figure 3, and the structure of the Al was fabricated using a CO_2_ laser with parameters for speed, density, and power. To measure the height and width of the cross-sectional morphology, the PDMS solution was poured into the MW-Al, and the resulting film was peeled off after curing in an oven at 85 °C for 1 h. The microstructure of the MW-Al and the corresponding PDMS film were then analyzed using optical microscopy (OM). As shown in Figure 3a,b, the surface of the MW-Al exhibited a grid-like shape with a regular hole array, with an average hole distance of approximately 250 µm. The cross-sectional morphology of the MW-Al, as shown in Figure 3c, reveals a wavy shape, with the height of the microwaves measuring about 18 µm and the wavy width measuring approximately 201.6 μm (see Figure 3d and Table 2).

**Figure 4:**
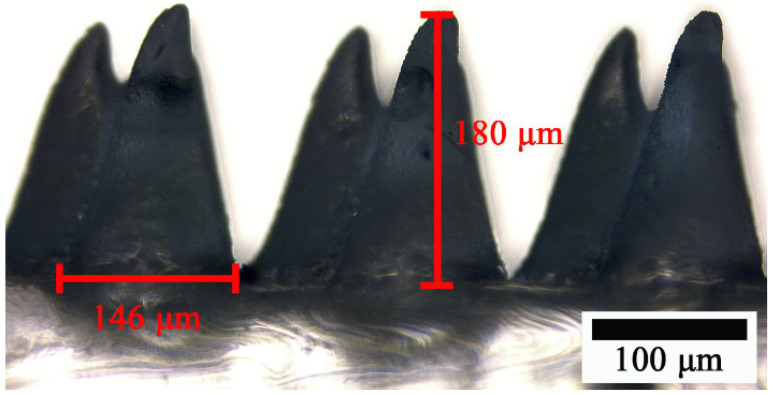
OM image of the microcone structure on PDMS.

The micromorphology of the MC structures on PDMS is displayed in Figure 4. The height and width of the microcones were measured using OM. The front-view image of the MC-PDMS reveals an average height of 180 µm, an average width of 146 µm, and a layer thickness of approximately 700 µm.

### 3.2. The Working Mechanism of the MW-MC-TENG

The working mechanism of the MW-MC-TENG can be explained as the combination of triboelectrification and electrostatic induction, where frictional materials and electrode materials play key roles, as shown in Figure 2a. Initially, when the two frictional materials are in full contact and rub against each other under external pressure, an equal number of transferred charges are generated on the surfaces of both materials, as shown in Figure 2b. According to the difference in the polarity of the triboelectric series, the MW-Al acquires positive charges, while the MC-PDMS gains negative charges. When the external pressure is released, the two charged layers begin to separate from each other. Due to the need for potential difference balance, the electrode flow is driven from the bottom electrode to the upper one, generating a current in the opposite direction, as shown in Figure 2c. When the two friction layers reach the maximum separation distance position, the flow of the electrons disappears for the electrostatic equilibrium, as shown in Figure 2d. If the external force is applied again, the opposite electrode flow is generated in the direction from the upper electrode to the bottom one due to the electrostatic induction, as shown in Figure 2e. When the friction layers re-contact, the induced charges of the opposite electrodes neutralize each other and reach an equal electrostatic equilibrium state. The periodic contact and separation between the MC-PDMS and MW-Al lead to charge transfer and redistribution on the contact surface.

### 3.3. The Output Performance of the MW-MC-TENG

**Figure 5:**
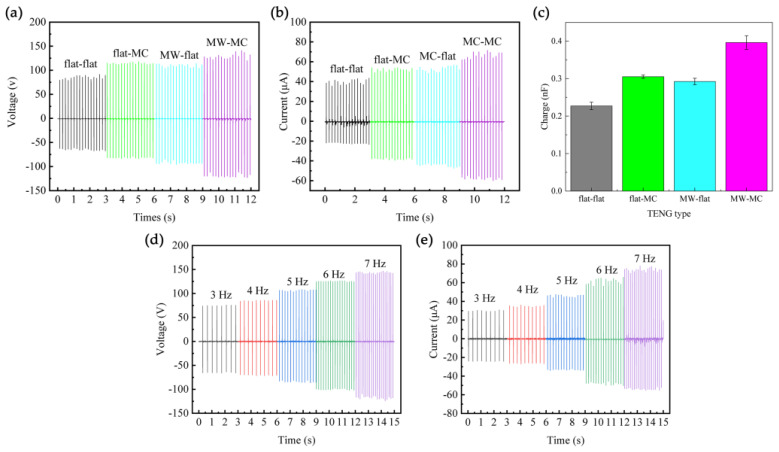
The electrical characteristic generated by the MW-MC-TENGs. (**a**–**c**) The open–circuit voltage, short–circuit current, and calculated transfer charge of the TENGs constructed by flat Al, flat PDMS, MW-Al, and MC-PDMS, respectively. (**d**,**e**) show the open-circuit voltage and the short-circuit current of the MW-MC-TENG under different contact-separation frequencies.

To compare the effect of the microstructure on the performance of the TENG, we prepared untreated Al (flat-Al) and untreated PDMS (flat-PDMS) to assemble TENGs alongside other test samples. The vertical pneumatic cylinder test platform was utilized to control the contact separation frequency (6 Hz). Figure 5a–c show the *V_oc_*, *I_sc_*, and *Q_sc_* of the TENGs with different friction pairs, demonstrating a trend as follows: flat-flat-TENG (91.5 V, 43.5 μA, 0.23 nF, 1.74 μA cm^−2^) < flat-MC-TENG (118 V, 54 μA, 0.31 nF, 2.16 μA cm^−2^) ≈ MW-flat-TENG (114 V, 56.5 μA, 0.29 nF, 2.26 μA cm^−2^) < MW-MC-TENG (141 V, 71.5 μA, 0.40 nF, 2.86 μA cm^−2^). The output performance of the flat-MC-TENG and MW-flat-TENG is higher than that of flat-flat-TENG, which can be attributed to the increased surface contact area provided by the roughened Al-flat PDMS combination or the flat Al-roughened PDMS combination. The double-side patterned MW-MC-TENG’s *V_oc_*, *I_sc_*, and *J_sc_* are approximately 54%, 64%, and 64% higher than those of the flat-flat-TENG without a microstructure. The output performance of the TENGs is significantly affected by the surface morphology of the friction pairs under different microstructure conditions. In total, the output performance of TENGs with roughened Al and PDMS friction layers is superior to that of other ones with more surface contact area. Consequently, the MW-Al and MC-PDMS were selected as the tribo-layers of MW-MC-TENG.

The output performance of the MW-MC-TENG was evaluated at different frequencies (3 Hz–7 Hz) using the testing platform, maintaining a constant distance of 20 mm (Figure 5d,e). The *V_oc_* and *I_sc_* gradually increase with the increase of contact-separation frequency and reach the peak value of 147 V and 78 μA, respectively, at the highest frequency. As the speed of contact and separation between the two friction layers increases, their contact time decreases, decreasing the number of neutral charges on the two plates. This phenomenon promotes an increase in both *V_oc_* and *I_sc_*.

### 3.4. Enhancement Mechanism of the MW-MC-TENG

**Figure 6:**
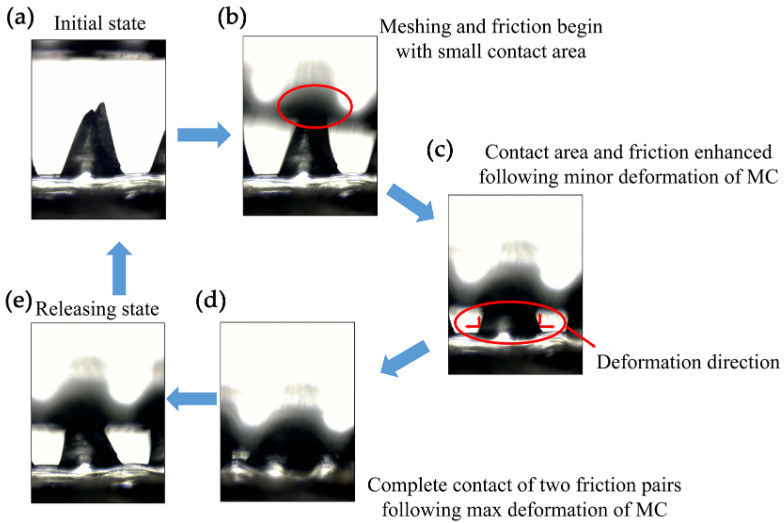
The enhancement process of the contact area and friction of the MCs in the meshing and deformation process. (**a**) The initial state of the MC-MW-TENG; (**b**) The starting contact point; (**c**) The contact area and friction increase with further meshing and deformation; (**d**) The multi-contact area and multi-friction between the MW-Al and MC-PDMS substrate at the maximum deformation state; (**e**) The releasing state.

Figure 6 illustrates the enhancement mechanism of the MW-MC-TENG through 3D modeling, depicting the meshing, deformation, and restoring process of the MCs during the cyclic pressing–releasing operation, as observed using OM. Figure 6a exhibits the initial state of the MC-MW-TENG, where no charging occurs. Figure 6b displays the initial contact state of the two friction pairs. At this state, MW-Al and MC-PDMS are not yet in contact with each other, and the slight deformation is primarily confined to the tip of the MCs. Figure 6c presents the further contact and friction state. In this state, the contact area increases with the increase in the deformation amount of the MC-PDMS, initiating dipole triboelectric charging. Figure 6d depicts the complete contact and deformation state, where the maximum equilibrium of triboelectric distribution is established between the two triboelectric materials. At this point, the contact area and deformation of the MC-PDMSs reached the maximum amount, which resulted in the high performance of the MW-MC-TENG and the peak of the voltage and current. Finally, Figure 6e illustrates the restoring state of the MC-PDMS after the external pressure is released, resulting in an unequal potential that generates a negative current through an external circuit.

It is evident from the OM–observation process that the morphology and surface area of the micropores in the Al film significantly impact the contact area of the two friction layers. In our case, the micropores in the MW-Al exhibit spherical crown shapes (see Figure 3a,c) with their dimensions listed in Table 3 and illustrated in Figure 3d. The average surface area of the micropores is calculated as 41,259.6 μm^2^, as indicated by Formula (1). Considering that the array density of micropores is 1600 #/cm^2^, the total number (N) of micropores is determined as 40,000, leading to a contact area of 2874.2 mm^2^, as derived from Formula (2), where L means the length of the square samples. It is evident that the contact area of the MW-MC pairs is considerably larger than that of flat-flat pairs, which have a contact area of 2500 mm^2^. Thus, the estimated and measured output electric performance is correspondingly higher. The measured voltages and currents are close to the calculated results.

S = 2πRH,(1)

CS = (L^2^ − Nπr^2^) + NS, (2)

**Figure 7:**
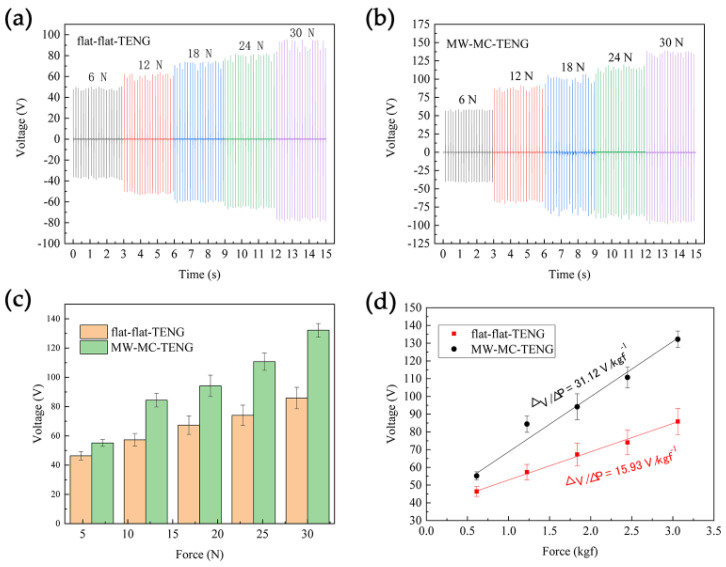
Output voltage details for (**a**) flat-flat-TENG, and (**b**) MW-MC-TENG under different forces. (**c**) The comparison of the two TENGs output voltage as a function of force. (**d**) The comparison of the two TENGs force sensitivity.

The deformation phenomenon shown in Figure 6 suggests the potential influence of the flexoelectric effect on the output performance of the TENG due to strain gradient. To further investigate and analyze the collaborative flexoelectric and triboelectric effects on the TENGs, the output voltage performance of the flat-flat-TENG and MW-MC-TENG was measured using a pushing platform tester. The flat-flat-TENG served as a prototype in which only the triboelectric effect contributed to the output voltage for comparison. The results shown in Figure 7a,b distinctly indicate the enhanced triboelectric charging in the MW-MC-TENG device; specifically, the output voltage of the MW-MC-TENG is greater than that of the flat device. Figure 7c illustrates that the difference in output between the double-side patterned and flat devices increases with applied force. Figure 7d shows that the force sensitivity of the MW-MC-TENG (31.12 V/kgf^−1^) is almost twice that of the flat-flat-TENG (15.93 V/kgf^−1^). This phenomenon is attributed to the increased polarization strength resulting from the flexoelectric effect as the MC deformation and strain gradient rise, leading to enhanced piezoelectric output and force sensitivity of the device. These results confirm the significant contribution of concurrent flexoelectricity in MW-MC-TENG.

### 3.5. Power Generation, Energy Storage, and Durability

**Figure 8:**
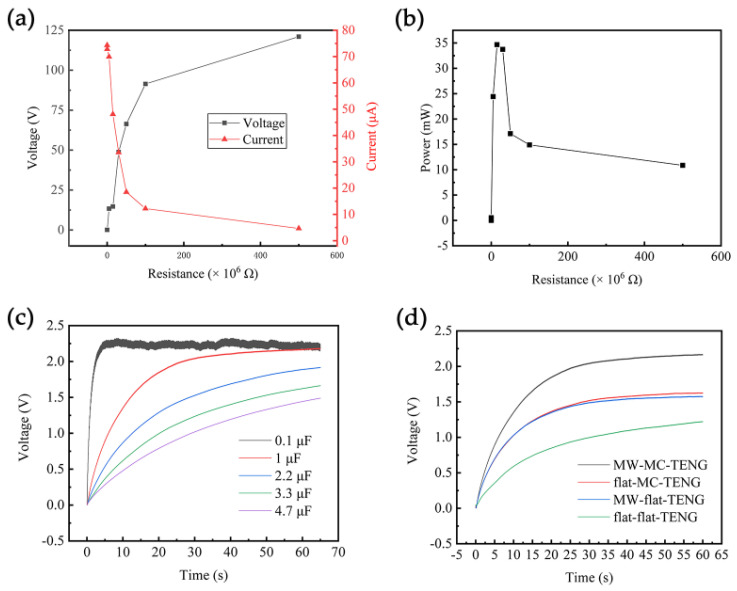
Output (**a**) voltage, current, and (**b**) power of the MW-MC-TENG under different external resistances. (**c**) Charging voltage of the MW-MC-TENG from 0.1 to 10 μF capacitor. (**d**) The charging voltage curve on a 1 μF capacitor of flat-flat-TENG, MW-flat-TENG, flat-MC-TENG, and MW-MC-TENG.

To achieve the maximum output power of MW-MC-TENG, it is externally connected to a range of resistors from 1000 Ω to 500 MΩ and tested with an external circuit. As the resistance value increases, the output voltage initially rises slowly and then rapidly (Figure 8a). In contrast, the output current exhibits a decreasing trend, transitioning from slow to fast. When the resistance is 15 MΩ, the voltage and current reach 14.7 V and 48.1 μA, respectively, resulting in a peak power of 34.7 mW (see Figure 8b) for the MW-MC-TENG, which corresponds to a power density of 1.4 mW/cm^2^.

Figure 8c illustrates the charging voltage of MW-MC-TENG as a function of time for six different capacitors (0.1–10 µF). The charging voltage (*V_c_*) reached 2.01 V at 2.28 s with a maximum value of 2.32 V for 0.1 µF. The 1 µF capacitor registered 1.80 V at 18.5 s, and the 2.2 µF capacitor recorded 1.7 V at 40.88 s. The 3.3 µF capacitor achieved 1.6 V at 57.29 s, and the 4.7 µF capacitor reached 1.4 V at 55.95 s. Furthermore, the charging behavior of the flat-flat-TENG, MW-flat-TENG, flat-MC-TENG, and MW-MC-TENG was tested for energy storage capabilities using the same 1 µF capacitor, as shown in Figure 8d. The MW-MC-TENG exhibited a stable *V_c_* of approximately 2.2 V, whereas the average *V_c_* for MW-flat-TENG and flat-MC-TENG was 1.6 V, and for the flat-flat-TENG, it was 1.2 V. The practice tests of electrical charging demonstrate the potential application of the power supply in electronic devices.

**Figure 9:**
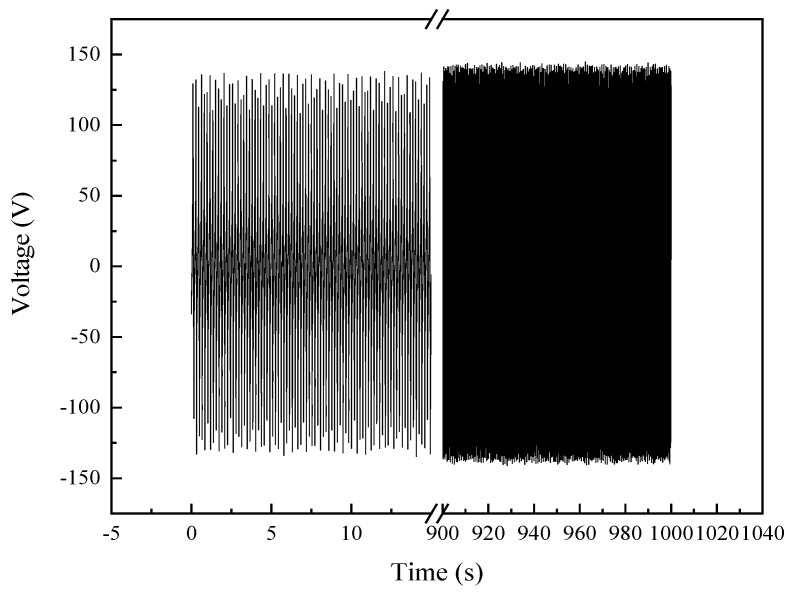
The electrical performance of MW-MC-TENG is stable after 6000 cycles of beating test.

The mechanical durability of the optimal TENG was confirmed, as it consistently generated electric power for 1000 s, corresponding to more than 6000 cycles of contact-separation operation, as shown in Figure 9.

### 3.6. Demonstration

**Figure 10:**
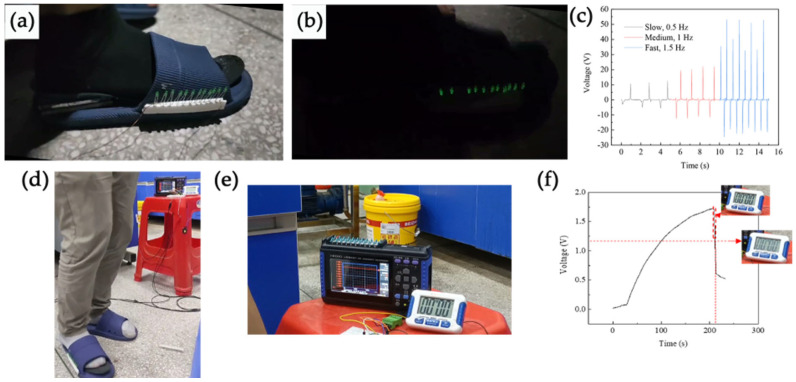
Application of the proposed TENG harvester. (**a**) Photographic image of the TENG device fixed into a shoe’s sole for harvesting the walking energy. (**b**) Eleven LEDs embedded in the shoe can be lit under medium walking speed. (**c**) The output voltage under different walking speeds. (**d**) The direct drive of the electronic timer by MW-MC-TENG through a 10 μF energy management circuit. (**e**,**f**) Shows the charging and driving process.

Since the MW-MC-TENG can generate high output voltage and power, it was tested for energy harvesting from human motion. The device was characterized under real-time human walking motions to evaluate its feasibility for daily life applications, specifically in supplying electricity for electronic devices. During testing, we embedded the MW-MC-TENG device within the insole of a shoe’s heel, as shown in Figure 10a. Figure 10b presents a snapshot of the instantaneous powering of 11 commercial LEDs driven by the proposed harvester prototype under a medium walking speed, demonstrating the potential application of MW-MC-TENG in self-powered luminous shoes for children. Figure 10c displays the output voltages at different walking speeds: 11 V, 20 V, and 55 V, indicating that the self-powered sensor exhibits high-speed sensitivity and can be applied for monitoring walking speed. Additionally, we compared our sensor with other devices, as shown in Table 4. The MW-MC-TENG-based insole sensor demonstrated higher output performance than other TENG sensors under the same walking/stepping speed or even slower.

To investigate the real-time applications of the harvester under practical conditions, we designed an energy management circuit by combining a rectifier bridge and a 10 μF capacitor. When the TENG is affixed to the insole of the shoes, it can continuously store electricity in the capacitor through stepping. As shown in Figure 10d,e, after stepping for nearly 180 s, and then turning on the switch, the electronic timer illuminated and lasted for 0.1 s.

## 4. Conclusions

In summary, this work presents a double-sided surface patterning method using CO_2_ laser technology, which is simple and cost-effective, to enhance the electrical output performance of TENGs. The MW-MC-TENG was fabricated by combining a PDMS featuring a microcone structure with an Al foil featuring a microwave structure. Two PMMA molds with the same micro-hole array were prepared through laser marking; the deeper mold was used to demold the PDMS, and the shallower one was used for cold imprinting the Al foil. The *V_oc_*, *I_sc_*, and *J_sc_* values of the MW-MC-TENG reached 141 V, 71.5 μA, and 2.86 μA/cm^2^, respectively, at a frequency of 6 Hz and a distance of 20 mm. When the resistance is 15 MΩ, the maximum power density of 1.4 mW/cm^2^ is achieved. Additionally, MW-MC-TENG can charge a 0.1 μF capacitor to 2.01 V in 2.28 s. Furthermore, the proposed TENG device can be also employed as a wearable insole to efficiently harvest walking energy, monitor step speed, and power a timer device. Based on these results, we believe that the proposed processing technique is essential for realizing an inexpensive triboelectric energy harvester and is advantageous for large-scale fabrication in future portable and wearable device applications.

## Figures and Tables

**Table 1:** Comparison of surface morphology, materials, fabrication methods, operation conditions, and electrical characteristics.

Morphology	Material	Fabrication Method	Operation Condition	Electrical Characteristics			Ref.
				*V_oc_* (V)	*I_sc_* (µA)	*Power Density* (mW/cm^2^)	
Microcone–microcone	PDMS@AgNWs-PDMS@ PTFE tiny burrs	Replication and molding, spray coating, evaporation and reactive ion etching	25 kPa, 0.6 Hz	3.14	0.0263	-	[29]
Yarn–yarn	TPU-PDMS	Coating	150 N, 6 Hz	76	3	~0.02	[30]
Nanogrit–nanogrit	PDMS@CNT-PDMS	Spin casting, dispersion and magnetic stirring	450 kPa, 1 Hz	92	55	0.007	[31]
Micropore–nanoparticles	Al/porous PDMS-Al@Au NPs	Spin coating and curing	50 N 10 Hz	~120	~0.125	-	[32]
Nano roughened structure–nanograting	PET-Au	Magnetron sputtering, inductively coupled plasma (ICP)	10 Hz	~125	~31.2	~0.32	[33]
Nanopillar–Nanopillar	Ni-PDMS	Spin coating, electrodeposition	10 kgf, 3 Hz	~100	~23	-	[34]
MW-Al and MC-PDMS	Al/PDMS/Al	CO_2_ laser ablation, cold imprinting	30 N 6 Hz	141	71.5	1.4	ours

**Table 2:** Detail of Types of TENG Devices for Each Combination.

Combination	Patterning Type	Structure of Al	Structure of PDMS	Electrical Characteristics		
				*V_oc_* (V)	*I_sc_* (µA)	*J* (µA/cm^2^)
flat-flat	Nonpatterned	Flat	Flat	91.5	43.5	1.74
flat-MC	Single-Sided	Flat	Microcone	118	54	2.16
MW-flat	Single-Sided	Microwave	Flat	114	56.5	2.26
MW-MC	Double-Sided	Microwave	Microcone	141	71.5	2.86

**Table 3:** The dimensions and area of the two types of Al friction layers.

Items	Symbol	MW-Al	Flat-Al
Size (cm^2^)		5 × 5	5 × 5
The square sample length (mm)	L	50	50
Average MW center depth (μm)	H	18	-
Average MW bottom diameter (μm)	d	201.6	-
Average MW bottom radius (μm)	r	100.8	-
Average MW spherical radius (μm)	R	365	-
Average MW estimated surface area (μm^2^)	S	41,259.6	-
Number of MWs	N	40,000	-
Grain density (number of MWs/cm^2^)		1600	-
Estimated surface area (Contact area) (mm^2^)	CS	2874.2	2500
Estimated voltage (V)		128.9	85.0
Estimated current (μA)		65.8	57.2
Measured voltage (V)		141	91.5
Measured current (μA)		71.5	43.5

**Table 4:** Comparison of the MW-MC-TENG device with other TENGs in output performance when used as an insole in stepping/walking motion monitoring application.

Device	Patterning Type	Area (mm^2^)	Frequency (Hz)	Voltage (V)	Ref.
P3000-T-TENG	Single-side	50 × 60	~4	~4	[41]
DSP-TiN/PDMS-based TENG	Single-side	40 × 40	~5	6	[42]
EC10S+PNy 11 TENG	Double-side	20 × 20	2	2	[43]
SF-TES	Double-side	20 × 20	~1.5	4	[44]
3D-FTENG	Double-side	28 × 30	1.5	40	[30]
MW-MC-TENG	Double-side	50 × 50	1.5	55	Ours

## Data Availability

Data are presented in the coauthors’ research results and schematic drawings available on request.

## References

[1] Wang S., Wang C.H., Yuan H.Z., Ji X.P., Yu G.X., Jia X.D. (2023). Size effect of piezoelectric energy harvester for road with high efficiency electrical properties. Appl. Energy.

[2] Wang Z.M., Chen Y.H., Jiang R.J., Du Y., Shi S.H., Zhang S.M., Yan Z.M., Lin Z.L., Tan T. (2023). Broadband omnidirectional piezoelectric-electromagnetic hybrid energy harvester for self-charged environmental and biometric sensing from human motion. Nano Energy.

[3] Bai S.M., Cui J., Zheng Y.Q., Li G., Liu T.S., Liu Y.B., Hao C.C., Xue C.Y. (2023). Electromagnetic-triboelectric energy harvester based on vibration-to-rotation conversion for human motion energy exploitation. Appl. Energy.

[4] Wan J., Wang H.B., Miao L.M., Chen X.X., Song Y., Guo H., Xu C., Ren Z.Y., Zhang H.X. (2020). A flexible hybridized electromagnetic-triboelectric nanogenerator and its application for 3D trajectory sensing. Nano Energy.

[5] Tu S., Tian T., Xiao T.X., Yao X.T., Shen S.C., Wu Y.S., Liu Y.L., Bing Z.S., Huang K., Knoll A. (2024). Humidity Stable Thermoelectric Hybrid Materials Toward a Self-Powered Triple Sensing System. Adv. Funct. Mater..

[6] Feng Z., Yang S., Jia S.X., Zhang Y.J., Jiang S., Yu L., Li R., Song G.W., Wang A.B., Martin T. (2020). Scalable, washable and lightweight triboelectric-energy-generating fibers by the thermal drawing process for industrial loom weaving. Nano Energy.

[7] Ryu H., Kim S.W. (2021). Emerging Pyroelectric Nanogenerators to Convert Thermal Energy into Electrical Energy. Small.

[8] Li H.Y., Bowen C.R., Yang Y. (2022). Phase transition enhanced pyroelectric nanogenerators for self-powered temperature sensors. Nano Energy.

[9] Ding R., Cao Z.Y., Wu Z.B., Xing H.T., Ye X.Y. (2021). All-in-One High Output Rotary Electrostatic Nanogenerators Based on Charge Pumping and Voltage Multiplying. ACS Nano.

[10] Rui P.S., Zhang W., Wang P.H. (2021). Super-Durable and Highly Efficient Electrostatic Induced Nanogenerator Circulation Network Initially Charged by a Triboelectric Nanogenerator for Harvesting Environmental Energy. ACS Nano.

[11] Wu Y.X., Li Y.S., Zou Y., Rao W., Gai Y.S., Xue J.T., Wu L., Qu X.C., Liu Y., Xu G.D. (2022). A multi-mode triboelectric nanogenerator for energy harvesting and biomedical monitoring. Nano Energy.

[12] He Y., Tian J., Peng W.B., Huang D.Y., Li F.P., He Y.N. (2023). On the contact electrification mechanism in semiconductor–semiconductor case by vertical contact-separation triboelectric nanogenerator. Nanotechnology.

[13] Yu Y., Gao Q., Zhang X.S., Zhao D., Xia X., Wang J.L., Li H.Y., Wang Z.L., Cheng T.H. (2023). Contact-sliding-separation mode triboelectric nanogenerator. Energy Environ. Sci..

[14] Ren D.H., Yang H.K., Zhang X.M., Li Q.Y., Yang Q.X., Li X.C., Ji P.Y., Yu P., Xi Y., Wang Z.L. (2023). Ultra-High DC and Low Impedance Output for Free-Standing Triboelectric Nanogenerator. Adv. Energy Mater..

[15] Wang Z.L., Zhu G., Yang Y., Wang S.H., Pan C.F. (2012). Progress in nanogenerators for portable electronics. Mater. Today.

[16] Cao X., Jie Y., Wang N., Wang Z.L. (2016). Triboelectric Nanogenerators Driven Self-Powered Electrochemical Processes for Energy and Environmental Science. Adv. Energy Mater..

[17] Wang Z.L. (2017). Catch wave power in floating nets. Nature.

[18] Wang Z.L., Chen J., Lin L. (2015). Progress in triboelectric nanogenerators as a new energy technology and self-powered sensors. Energy Environ. Sci..

[19] Liu X.R., Zhao Z.H., Gao Y.K., Nan Y., Hu Y.X., Guo Z.T., Qiao W.Y., Wang J., Zhou L.L., Wang Z.L. (2024). Triboelectric nanogenerators exhibiting ultrahigh charge density and energy density. Energy Environ. Sci..

[20] Li Z.J., Yang C., Zhang Q., Chen G., Xu J.Y., Peng Y., Guo H.Y. (2023). Standardized Volume Power Density Boost in Frequency-Up Converted Contact-Separation Mode Triboelectric Nanogenerators. Research.

[21] Guess M., Soltis I., Rigo B., Zavanelli N., Kapasi S., Kim H., Yeo W.-H. (2023). Wireless batteryless soft sensors for ambulatory cardiovascular health monitoring. Soft Sci..

[22] Lin L., Xie Y.N., Niu S.M., Wang S.H., Yang P.K., Wang Z.L. (2015). Robust triboelectric nanogenerator based on rolling electrification and electrostatic induction at an instantaneous energy conversion efficiency of ~55%. ACS Nano.

[23] Kim D., Lee H.M., Choi Y.K. (2017). Large-sized sandpaper coated with solution-processed aluminum for a triboelectric nanogenerator with reliable durability. RSC Adv..

[24] Zi Y.L., Niu S.M., Wang J., Wen Z., Tang W., Wang Z.L. (2015). Standards and figure-of-merits for quantifying the performance of triboelectric nanogenerators. Nat. Commun..

[25] Fan F.R., Lin L., Zhu G., Wu W.Z., Zhang R., Wang Z.L. (2012). Transparent Triboelectric Nanogenerators and Self-Powered Pressure Sensors Based on Micropatterned Plastic Films. Nano Lett..

[26] Cheng G.G., Jiang S.Y., Li K., Zhang Z.Q., Wang Y., Yuan N.Y., Ding J.N., Zhang W. (2017). Effect of argon plasma treatment on the output performance of triboelectric nanogenerator. Appl. Surf. Sci..

[27] Deng W.L., Zhang B.B., Jin L., Chen Y.Q., Chu W.J., Zhang H.T., Zhu M.H., Yang W.Q. (2017). Enhanced performance of ZnO microballoon arrays for a triboelectric nanogenerator. Nanotechnology.

[28] Trinh V.L., Chung C.K. (2017). A Facile Method and Novel Mechanism Using Microneedle-Structured PDMS for Triboelectric Generator Applications. Small.

[29] Yao G., Xu L., Cheng X.W., Li Y.Y., Huang X., Guo W., Liu S.Y., Wang Z.L., Wu H. (2020). Bioinspired Triboelectric Nanogenerators as Self-Powered Electronic Skin for Robotic Tactile Sensing. Adv. Funct. Mater..

[30] Li M.Q., Xu B.A., Li Z.H., Gao Y.Y., Yang Y.J., Huang X.X. (2022). Toward 3D double-electrode textile triboelectric nanogenerators for wearable biomechanical energy harvesting and sensing. Chem. Eng. J..

[31] Rasel M.S., Maharjan P., Salauddin M., Rahman M.T., Cho H.O., Kim J.W., Park J.Y. (2018). An impedance tunable and highly efficient triboelectric nanogenerator for large-scale, ultra-sensitive pressure sensing applications. Nano Energy.

[32] Chun J.S., Ye B.U., Lee J.W., Choi D., Kang C.Y., Kim S.W., Wang Z.L., Baik J.M. (2016). Boosted output performance of triboelectric nanogenerator via electric double layer effect. Nat. Commun..

[33] Wang H.S., Jeong C.K., Seo M.H., Joe D.J., Han J.H., Yoon J.B., Lee K.J. (2017). Performance-enhanced triboelectric nanogenerator enabled by wafer-scale nanogrates of multistep pattern downscaling. Nano Energy.

[34] Choi H.J., Lee J.H., Jun J., Kim T.Y., Kim S.W., Lee H. (2016). High-performance triboelectric nanogenerators with artificially well-tailored interlocked interfaces. Nano Energy.

[35] Cai X., Xiao Y., Zhang B.W., Yang Y.H., Wang J., Chen H.M., Shen G.Z. (2023). Surface Control and Electrical Tuning of MXene Electrode for Flexible Self-Powered Human–Machine Interaction. Adv. Funct. Mater..

[36] Lin L., Chung C.K. (2021). PDMS Microfabrication and Design for Microfluidics and Sustainable Energy Application: Review. Micromachines.

[37] Zhu S.L., Xia Y.F., Zhu Y., Wu M., Jia C.Y., Wang X. (2022). High-performance triboelectric nanogenerator powered flexible electroluminescence devices based on patterned laser-induced copper electrodes for visualized information interaction. Nano Energy.

[38] Ke K.H., Lin L., Chung C.K. (2022). Low-cost micro-graphite doped polydimethylsiloxane composite film for enhancement of mechanical-to-electrical energy conversion with aluminum and its application. J. Taiwan Inst. Chem. Eng..

[39] Trinh V.L., Chung C.K. (2018). Harvesting mechanical energy, storage, and lighting using a novel PDMS based triboelectric generator with inclined wall arrays and micro-topping structure. Appl. Energ..

[40] Chung C.K., Ke K.H. (2020). High contact surface area enhanced Al/PDMS triboelectric nanogenerator using novel overlapped microneedle arrays and its application to lighting and self-powered devices. Appl. Surf. Sci..

[41] Song J., Gao L.B., Tao X.M., Li L.X. (2018). Ultra-Flexible and Large-Area Textile-Based Triboelectric Nanogenerators with a Sandpaper-Induced Surface Microstructure. Materials.

[42] Xiao Y., Lv X., Yang L.P., Niu M.Y., Liu J.C. (2021). A High-Performance Flexible Triboelectric Nanogenerator Based on Double-Sided Patterned TiN/PDMS Composite Film for Human Energy Harvesting. Energy Technol..

[43] Prasad G., Graham S.A., Yu J.S., Kim H.D., Lee D.W. (2023). Investigated a PLL surface-modified Nylon 11 electrospun as a highly tribo-positive frictional layer to enhance output performance of triboelectric nanogenerators and self-powered wearable sensors. Nano Energy.

[44] Gajula P., Muhammad F.M., Reza M.S., Jaisankar S.N., Kim K.J., Kim H.D. (2023). Fabrication of a silicon elastomer-based self-powered flexible triboelectric sensor for wearable energy harvesting and biomedical applications. ACS Appl. Electron. Mater..

